# Supplementary material for: Characteristics and Service Utilization by Complex Chronic and Advanced Chronic Patients in Catalonia: A Retrospective Seven-Year Cohort-Based Study of an Implemented Chronic Care Program
Source: Int J Environ Res Public Health. 2021 Sep 8;18(18):9473. doi: 10.3390/ijerph18189473 (PMC8464881; doi:10.3390/ijerph18189473)
Supplement: Supplementary file 1 [file ijerph-18-09473-s001.zip › ijerph-1296675-supplementary.pdf]

Supplementary File for the submission by Santaeugènia et al. "Characteristics and Service Utilization by Complex Chronic and Advanced Chronic Patients in Catalonia: A Retrospective Seven-year Cohort-Based Study of an Implemented Chronic Care Program."

Supplementary Tables

Table S1. Types of complexity and criteria for the identification of complex chronic patients

**Type of Complexity**	**Definition**	**Criteria[a]**
**Clinical Complexity**	Interaction between multiple disease-related factors.<br>E.g. Diagnosis, severity, symptoms with difficult control, multimorbidity, and associated disability degree.<br><br>For healthcare professionals, disease-related factors usually influence two interrelated conditions, difficulty for accurate diagnosis and for decision-making, determining clinical complexity.	▪ Multimorbidity: Single Chronic severe or progressive pathology. Rare diseases.<br>▪ Dynamic evolution.<br>▪ Geriatric syndromes with severity/progression criteria (e.g., polypharmacy, cognitive determinant, and frailty).<br>▪ Intense, persistent and refractory symptoms.<br>▪ High services use and healthcare resource consumption. High probability of decompensation.<br>▪ Classification within the 5% higher risk according to the Adjusted morbidity group.
**Contextual complexity** (social, psychosocial and familiar)	Disease-generated social needs and psychosocial discomfort that may worsen or hamper management of clinical complexity.<br>Risky social function (i.e., dysfunctional) to meet health needs within the same social support network due to the impact of social and psychosocial risk factors.	Risky or dysfunctional social situation to meet needs in one or more of the following areas:<br>▪ Organization of care.<br>▪ Family unit and/or caregivers.<br>▪ Family cohesion and affective climate.<br>▪ Structural, safety, comfort and privacy conditions.<br>▪ Social and setting network.
**Complexity associated with the healthcare system**	Given the multiple possible responses to the complexity challenge, paradoxically, the system may become a source of complexity, not necessarily solved by increased resources and services. Increased services and healthcare professionals may lead to complexity due to the generation of multiple possible	▪ Benefit of multidisciplinary management. Need to access different services.<br>▪ Uncertainty in decision-making and doubts regarding management. Discrepancies among different professionals or teams regarding clinical management.

	pathways, uncoordinated responses, duplicities, and gaps in the healthcare continuum.	▪ Benefit of integrated care strategies.
[a]unequivocal guidelines regarding criteria, their number, or combinations that need to be met to be considered a chronic complex patient have not been established. Healthcare professionals may deem management of a case as particularly difficult due to a sufficient number of criteria met, validate them clinically, and identify the patient as chronic complex. A person failing to meet any of the criteria is unlikely to have complex care needs.		

Supplementary Figures

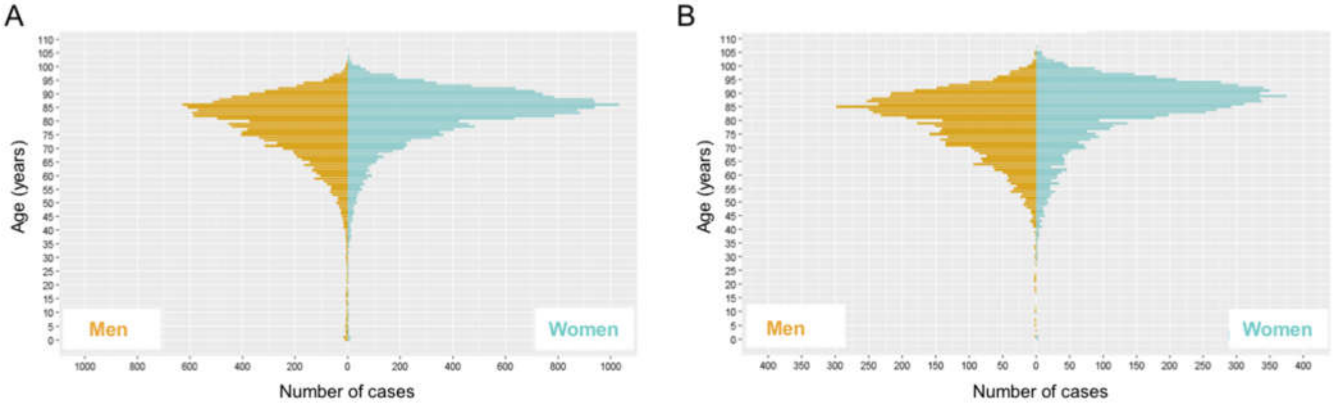

Figure S1. Age and sex distribution of prevalent cases of (A) CCPs and (B) ACPs in 2019.

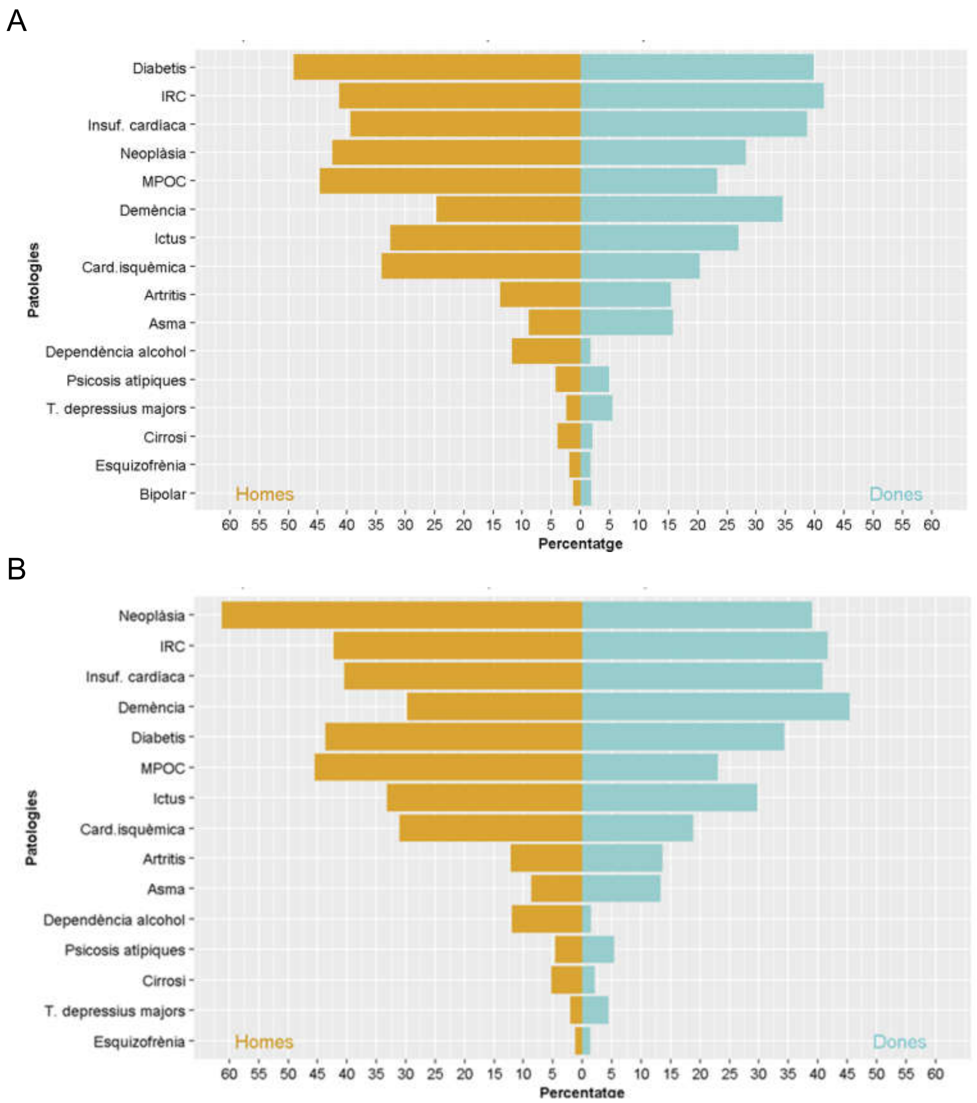

Figure S2. Main comorbidities of prevalent cases of (A) complex chronic (CCP) and (B) advanced chronic (ACP) patients by sex. Year 2019.
